# When a mother has cancer: strains and resources of affected families from the mother’s and father’s perspective - a qualitative study

**DOI:** 10.1186/s12905-018-0562-8

**Published:** 2018-05-25

**Authors:** Laura Inhestern, Corinna Bergelt

**Affiliations:** Department of Medical Psychology, University Medical Center Hamburg-Eppendorf, Martinistr. 52, 20246 Hamburg, Germany

**Keywords:** Mother, Cancer, Family, Children, Resources

## Abstract

**Background:**

When a mother has cancer, families with minor children are confronted with major challenges for all family members. According to the Family Adjustment and Adaptation Response (FAAR) Model, the (im) balance between strains and resources of families affected by cancer can be an important indicator on the families’ adjustment to the situation. Hence, this study aims to explore the strains and resources of families of mothers with cancer from the mother’s and father’s perspective.

**Methods:**

We conducted semi-structured interviews with 29 mothers diagnosed with cancer and ten fathers. The data was transcribed verbatim and analyzed using thematic analysis.

**Results:**

Both, mothers and fathers, reported a general impact of the disease regarding social and practical changes as well as strong emotional reactions. Parents reported specific strains and stressors regarding their parental role e.g. changes in the self-concept as a parent or fears and concerns about the children. Many mothers additionally experienced feelings of guilt. All fathers reported an increase of responsibilities and pressure. Both, the ill and healthy parent, reported strains and stressors for their children, e.g. parents observed behavioral changes and strong emotional reactions in their children. Families used a variety of resources and coping strategies on external, family and intrapersonal levels to encounter the challenges of the disease. They reported that e.g. support networks, flexible working hours and competent medical staff were helpful. Moreover, on the family level e.g. family time, open communication and the children themselves were considered to be important resources. On the intrapersonal level, parents reported resources such as setting small aims for the future and taking time for oneself.

**Conclusions:**

Our findings indicate a high amount and diversity of stressors and strains for the ill and healthy parent and for their children. At the same time, parents use diverse resources and coping strategies on external, family or intrapersonal level. The assessment of strains and resources may be an important indicator for the support needs of families when a mother has cancer. Enhancing and activating resources and coping strategies may help the families to manage the situation better and may prevent maladjustment in the family members.

## Background

Estimates suggest that about 18% of cancer patients have minor children [1]. Patients experience the adverse effects of illness, treatment and emotional consequences of a life-threatening diagnosis [2, 3]. As parents, they are caring for their dependent children at the same time. Parents affected by cancer have to deal with concerns regarding their children, communicating with their children about cancer or feelings of guilt [4–6]. Additionally, all family members live through a challenging time affected by changes and disruption in family life and routines. Children can experience high levels of stress and can develop emotional and behavioral problems [7, 8].

Based on the family adjustment and adaption response model (FAAR model) [9], a major stressor such as parental cancer can lead to an imbalance between family members’ strains, stressors and daily hassles and their capability to handle the situation, and hence, may lead to adverse emotional outcomes in parents or children [9]. To respond successfully to the challenge of a cancer diagnosis, families need strengths and resources, which can help to “rally in times of crisis, to buffer stress, reduce the risk of dysfunction, and support optimal adaptation” [10]. Studies identified resources such as individual strengths and social support [11, 12] or certain communication patterns within the family [13, 14]. Unfortunately, research on parental cancer has so far mainly focused on the strains and stressors or the emotional impact on either the ill parent or the children while resources and coping strategies of the family members have not been systematically investigated yet. Studies including both the mothers’ and the fathers’ perspectives are rare [15] and mainly focus on parent-child communication about cancer [16–18] or on changes in child-care [6].

However, the experiences of both parents (ill and healthy) and the capabilities of families to deal with the situation may have a great impact on the adaptation of the family members to parental cancer. Hence, the aim of this study was to explore the cancer-specific strains and stressors of family members and families as well as resources and coping strategies from the perspective of both mothers and fathers.

## Method

### Study design

We conducted a qualitative study using semi-structured interviews to explore the strains and resources of families and used thematic analyses to systemize the findings. The interviews were conducted using an interview guideline, which was developed based on prior literature, clinical experience and the study aims. This study was reviewed and approved by the local ethics committee of the Chamber of Psychotherapists of Hamburg, Germany, in December 2014.

### Participants

To capture the variation across the course of the cancer disease and its treatment, we included a heterogeneous sample regarding time since diagnosis, treatment (current versus post-treatment) and family variables (single parent versus cohabitation) [19]. Women with cancer were recruited consecutively on several wards of a University Cancer Center Hamburg (UCCH), in an inpatient rehabilitation clinic and in an outpatient counselling service. They were eligible if they had at least one child < 18 years, were German speaking and physically and mentally able to conduct an interview. Medical staff or psychologists of the cooperating institutions identified eligible participants and the researcher (LI) invited them to participate. Prior to the interview, mothers received written information on the study aims and gave their informed consent.

As we aimed at interviewing mothers diagnosed with cancer and, if applicable, the healthy fathers, an additional inclusion criterion for the fathers was the patient’s consent to contact the father. If the mothers gave their consent, the healthy father was contacted face-to-face if he attended the cooperating institution or via phone. Fathers also received written information on the study aims and were invited to participate. Again, informed consent was obtained before conducting the interviews.

### Data collection

We used a semi-structured interview guideline (Table 1), which allows individualized questions in order to explore the experience of the interviewees. All interviews were conducted between April and December 2015 by the first author, LI (psychologist, PhD candidate), who is experienced in qualitative research. At the beginning of each interview, LI introduced herself as a junior researcher with the research focus on parental cancer in the psychooncological research group of the Institute of Medical Psychology and gave information about the study. The first interview was a pilot interview to test and redefine the guideline. Since we conducted only minor changes in the guideline, the pilot interview was included in the final data analyses. We continued recruitment of ill mothers until no new themes derived from the interviews of the ill mothers and data saturation was reached [20].

**Table 1:** Overview of the interview items for mother and fathers

Central questions	Check
How was the course of the disease so far?	□Diagnosis
	□Progress
	□Symptoms
	□Treatment
	□Side effects
What changes, stressors and challenges do you face because of the disease?	□myself
	□as a parent
	□my partner
	□children
	□as a family
Can you describe what kind of support you would have needed or still need with regard to your parental role?	□personal needs
	□needs of family members
	□use of support system
What did help you and your family during the course of the disease?	□resources
	□sources of strength as a parent
	□sources of strength for the family

We conducted 29 interviews with ill mothers and ten interviews with fathers. Eight women were single parents; 11 mothers did not give the consent to contact their partner. Reasons were mainly to prevent them from additional demands and duties.

The mean duration of the interviews was 40 min (Range: 18 to 95 min). Field notes were taken during the interviews. To avoid a selection bias regarding organizational issues, mothers and fathers could choose to be interviewed during inpatient clinic stays, at home or by telephone. Correspondingly, the interviews took place at the wards or offices of the cancer center, at an office of the rehabilitation clinic or by phone. At the wards of the cancer center other patients were present in the same room in some cases, but it was possible to maintain privacy due to the arrangement in the room nevertheless. In two families, both parents were interviewed simultaneously, because the families could not arrange two appointments. All interviewees gave their informed consent to be interviewed and for the interview to be recorded, transcribed verbatim and analyzed for the study.

### Data analysis

The transcripts were analyzed with thematic analysis based on the approach of Braun and Clarke [21] using the qualitative data analysis software MaxQDA. Transcripts were not returned to the interviewees for corrections or feedback.

The interviews of mothers and fathers were analyzed separately from each other and accordingly, findings were aggregated separately for mothers and fathers. Following the approach of thematic analysis, the first phase of data analyses was to familiarize with the data by reading the transcripts repeatedly (LI and a student assistant). In the second phase, segments of the data regarding ‘demands and capabilities of the families’ were extracted. Based on the FAAR Model demands included stressors, strains and daily hassles and capabilities included resources and coping behaviors [9]. The next phase consisted of organizing the extracted codes to descriptive themes and subthemes inductively. A review of the fit between extracted codes and themes/subthemes was performed by two independent researchers (LI and a student assistant for about 50% of the material (*n* = 20). The agreement between the two researchers after this phase was 83%. If necessary, themes or subthemes were redefined by consensus and eventually themes and subthemes were finalized. After that, one researcher (LI) again analyzed the entire material with regard to the final themes and subthemes and selected illustrative codings.

## Results

### Sample characteristics

A total of 29 ill mothers and 10 fathers from 29 families were interviewed (Table 2). Eight mothers were single parent. Mean age of ill mothers was 39.4 years (range 30–51); mean age of fathers was 37.6 years (range 32–49). Twenty women were diagnosed with breast cancer and mean time since diagnosis was 21.3 months (range 2–77). The 29 families had 45 children in total; mean age of the children was 7.4 years. In 17 families the youngest child was 5 years or younger.

**Table 2:** Characteristics of interviewed parents (*n* = 39)

Characteristics	Number
Patients (*n* = 29)	
Age (years; M, Range)	39.4 (30–51)
Family status	
Living with the partner/other parent	21
Single Parent	8
Education	
≤ 11 years	13
12–13 years	16
Job qualification	
Three-year training	16
University degree	10
other	3
Main earner of the family	11
Time since initial diagnosis (months; M, Range)	21.3 (2–77)
Diagnosis	
Breast cancer	20
Other (e.g. leukaemia, gynaecological tumour, tumour of respiratory system)	9
Number of children	
1	13
2	16
Age of children (years; M, Range)	7.4 (1–29)
Age of youngest child	
≤ 5 years	17
6–12 years	10
> 12 years	2
Children	
Male	27
Female	18
Partners (*n* = 10)	
Age (years; M, Range)	37.6 (32–49)
Education	
≤ 11 years	2
12–13 years	8
Job qualification	
Three-year training	3
University degree	7
Other	–
Main earner of the family	7

### Demands – strains and stressors

#### General impact of cancer

One major theme the participants elaborated on during the interviews was the general impact of cancer diagnosis and treatment (Tables 3 and 4). All interviewed mothers and fathers reported immediate consequences of diagnosis and treatment. Mothers as well as fathers reported that they were shocked after diagnosis. Ill mothers experienced side effects of treatment such as nausea, pain or fatigue. For most fathers it was difficult to see and deal with their partner’s suffering due to the cancer and its treatment.

**Table 3:** Themes and subthemes regarding demands and capabilites of mothers affected by cancer (*n* = 29) and healthy fathers (*n* = 10)

	Mothers with cancer (*n* = 29)	Healthy fathers (*n* = 10)
Demands		
General impact of cancer		
Immediate consequences of diagnosis and treatment (e.g. shock of diagnosis, effects of treatment and diagnosis)	29	10
Practical and social changes (e.g. temporary job changes, daily routines, loss of friends)	27	10
Strong emotions (e.g. loss of control, fears, uncertainty)	28	7
Strains/Stressors regarding the parental role		
Changes of the self-concept as a parent (e.g. other family roles, attachment)	20	9
Feelings of guilt	18	1
Fears and concerns about the children (e.g. emotional consequences for children)	26	7
Communication about the disease	18	7
Increase of responsibilities and pressure (e.g. balancing multiple needs, work, time for kids)	10	10
Strains/stressors for the children		
Confrontation with disease and consequences (e.g. physical changes of mother, treatment)	22	9
Behavioral changes (e.g. school problems, withdrawal)	23	6
Fears and concerns/strong emotions (e.g. uncertainty, fear to lose the mother)	20	3
Seeing the vulnerability of the parents (e.g. parents crying, parents being helpless)	21	4
Loss of normal life and activities (e.g. daily routines, temporary loss of closest attachment figure)	15	4
Capabilities		
External resources		
Support network (e.g. friends, peers, school)	29	10
Competent medical staff	12	4
Flexible working hours	7	9
Financial security	3	–
Children’s books or broschures	7	–
Families resources and coping strategies		
Family time (e.g. routines, activities)	26	9
Children themselves	24	9
Open communication	18	5
Maintaining daily life	15	7
Emotional support from partner and family/strong relationship	12	3
Family cohesion	9	5
Flexible role division	8	5
Intrapersonal resources and coping strategies		
Time for oneself	21	4
Small aims for the future	10	5
Self-efficacy	10	2
Optimism/Positive attitude	9	4
Spirituality	5	–

**Table 4:** Strains and stressors of families when a mother has cancer including illustrative quotes

Themes	Quotes^a^
General impact of the disease	
Immediate consequences of diagnosis and treatment	• *“I am sensitive to everything I have to take. My body has completely rebelled. I threw up and felt so miserable. I fully collapsed.”* (Single mother of two children)
Practical and social changes	• *“Well, at the moment it’s like you just can’t go on as before. Of course you do everything a bit slower or you just leave things undone. You tell yourself that it’s just not possible right now.”* (Mother of two sons)
Strong emotions	• *“You get depressed. At least I did. I was never like this. I was always a fun-loving person. But eventually it catches up with you and you ask yourself why.”* (Mother of two children)
Strains and stressors regarding the parental role	
Changes of the self-concept as a parent (e.g. other family roles, attachment)	• *“My child is still there and makes demands. I am quickly irritated. I still have the same expectations even though I keep trying to lower them. It’s frustrating not being able to manage [everything] as before.”* (Mother of one daughter) • *“Certainly, one way or another, there has been a shift […], when I don’t have to work […], when you say ‘let’s go to the zoo or ride our bikes by ourselves’. So my wife can relax for half a day or at least for a couple of hours. Certainly, this has shifted […].”* (Father of one daughter)
Feelings of guilt	• *“I am so sorry that they have a sick mother. But I do the best I can.”* (Single mother of two children)
Fears and concerns about the children (e.g. emotional consequences for children)	• *“The worst thing for me was to see my child every day and to think that I will not be able to watch her grow up. Psychologically, this was the worst – the constant fear that I won’t be able to watch her grow up because she was so young.”* (Mother of one daughter) • *“But as far as I can see, for now it’s working quite well. While I am not worried at the moment I certainly do pay attention. Especially to him since he is a little older. I’d say that it is normal that he thinks and sometimes asks a question about it. I just want to make sure that he does not become too preoccupied.”* (Father of two children)
Communication about the disease	• *“Especially stressful was to figure out what to say. It still bothers me a bit. What do we say, how do we say it, or should we say anything at all. He came to the hospital, so he knew that I was sick and had surgery. We decided against telling him the whole truth. Since I was unable to lift him, we told him that I had a bad arm. ‘Mom can’t lift you up because she has bad arm. And to make sure she gets well we are here and do exercises and other things.’ I think this is okay. He accepted it. As I said, I really did not want to tell my 4-year-old exactly what is wrong with me.”* (Mother of one son) • *“Seeing my wife suffer hurt me. It also became increasingly more difficult to explain to the children ‘mom is not doing well and has to sleep.’ So it was good that therapy ended after 6 treatments.”* (Father of two children)
Increase of responsibilities and pressure (e.g. balancing multiple needs, work, time for kids)	• *Then I spent a lot of time at home and tried to deal with him as usual. Even when I was not doing well I tried to hide it and not allow him to see my pain.* (Single mother of one son) • *“When one parent drops out, you become solely responsible. You have to change the diapers, tuck them in at night, cook,* etc. *I think that this fact caused the intensity [of responsibilities] to increase.”* (Father of two children)
Additional burden of single mothers	• *“To me it is important, to consider the constellation of the family. It is a huge difference to be in a partnership or not. And if being single parent, one needs to consider the relationship between the ex-partners. In my case it is horrible and an additional stressor.”* (Single mother of one son) • *“He (ex-partner, father of the children) thinks he should take away my children. And that I am the reason for him (the son) having difficulties at school.”* (Single mother of two children)
Impact on the children (Mothers’ and fathers’ perspective)	
Confrontation with disease and consequences	• *“The oldest also had a hard time dealing with the illness. You could tell that she was thinking about it a lot. She will turn 7 in September. At times she would become very quiet.”* (Single mother of two children)
Behavioral changes	• *“My son’s difficulties became apparent at school. Now it is obvious that he has issues.”* (Single mother of two children)
Fears and concerns/strong emotions	• *“Although it has faded a bit, this autumn he had quite fears of abandonment. He was clinging, he was afraid to loose me.”* (Mother of two children)
Seeing the vulnerability of the parents	• *“I think he becomes irritable at times because he sees me as being weak. I was supposed to be his rock […] he has seen me cry and being desperate. He has not before. Then I wonder how he can take it. This could be a burden for him.”* (Mother of one son)
Loss of normal life and activities	• *“I also believe, even though he won’t admit it, […] that he misses my mothering, my taking care of him – definitely.”* (Mother of two sons)

^a^original transcripts in German, quotes have been translated

Almost all participants experienced practical and social changes in their daily lives. For ill mothers, routines such as household chores or child-care duties were more exhausting, and mothers reported they felt that the need to learn to let things go. All fathers had to take up more daily duties during the treatment period. As most fathers were the principal earner of the families, they simultaneously had to fulfil their occupational obligations and to take care of the children and their ill partner. This led to multiple burdens, which most of the fathers took as a challenge and countered pragmatically.

Both mothers and fathers experienced strong emotions such as fears concerning the uncertainty of diagnosis and treatment*.* Some fathers reported difficulties in coping with the disease, but trying to be strong for their family and to hide their fears and concerns.

#### Strains and stressors regarding the parental role

With regard to their parental roles ill mothers and healthy fathers experienced several strains and stressors such as changes regarding their self-concept as a parent. Many mothers struggled with their identity as a mother and described falling short of their expectations of being a good mother. In particular, mothers of young children reported they had to adjust their expectations with regard to breast feeding and being the closest attachment figure. Other mothers felt not being an integral part of the family anymore, but being ‘the ill one’. Fathers had to adjust their self-concept and had to redefine their role as a father. In almost all families, the mother had been the primary attachment figure prior to the diagnosis and it was a new experience for some fathers to take care for the children all alone temporarily.

Many mothers experienced feelings of guilt towards their children. They longed for spending more time with their children and being more attentive to them. Mothers felt guilty that their children had to experience their mother’s disease. Some mothers felt that they had lost their ease in interacting with their children due to increased irritability and inner distance to their children.

Most mothers and fathers reported that they were concerned about the children. They expressed fears regarding the possible heritability of the disease and potential negative psychological consequences for their children. In some cases, where the mothers were diagnosed with advanced cancer, parents had to confront the possibility of the mother’s death and feared that their children would become half-orphans.

Both mothers and fathers reported being insecure about what and how to talk with their children about their disease. Mothers struggled to find age-appropriate ways to talk to their children about their disease, but most of them finally decided to disclose their condition. However, some mothers did not disclose entirely and withheld information from the children. Some fathers reported ambivalent feelings, since they wanted to protect their children from the disease but at the same time felt that they should openly talk with them about it. They were worried about their children’s ability to cope with the situation and felt insecure about how much to disclose.

All fathers and some mothers reported increased responsibilities or pressure. Balancing the needs of the children and their own needs as a patient was difficult for some mothers. They felt a high pressure to recover quickly and to get back to daily routines as soon as possible for the sake of their children. While suffering physically and emotionally, mothers tried to maintain the impression of being well in front of their children. Most fathers expressed that they felt strained by the necessity to be the parent who was still functioning and thus being responsible to fulfil all duties. In consequence, they felt that they could not allow themselves to break down.

#### Additional burden of single parents

The eight single mothers reported several aspects which additionally affected them due to their single-parenthood. One aspect was that some single mothers missed a partner who could support them emotionally during the disease. Several single mothers disagreed with the fathers on what to tell the children and had conflicts about splitting times of child-care. Moreover, single mothers reported having little time to care for themselves or time to rest to recover physically from treatment.

#### Strains and stressors of the children

Both fathers and mothers reported several implications of the disease for their children (Tables 3 and 4). Most parents reported their children were challenged by the disease and its consequences e.g. the physical changes of their mother or the treatment. Mothers and fathers observed different coping strategies in their children. Some reported that their children withdrew and did not want to talk about the situation while others reported that their children asked many questions and tried to comprehend the situation. Many mothers and fathers observed behavioral changes in their children. Some children were seeking more attention, while others acted rebellious and defiant. Some mothers and fathers experienced increased maturity and seriousness in their children. Rarely, parents described school problems or that their child would imitate the physical symptoms of the mother.

With regard to the disease and its treatment mothers and fathers described that their children struggled seeing the vulnerability of their parents. Many mothers and some fathers reported that seeing the parents crying and helpless or perceiving physical weakness in their mother stressed the children. While some children responded to physical and emotional changes in their mother with incomprehension and anger, other children reacted compassionately.

Many mothers and fathers observed fears in their children, which they attributed to the disease. The fears mainly referred to separation, loss or uncertainty. In some families, the children were concerned about the well-being of their mother.

According to the parents, many children experienced losses e.g. of normalcy in daily life or activities during the course of disease. For the children the hospital stays of the mother often were the first time of separation from their mother for a prolonged period. Moreover, the children experienced reduced attentiveness and availability of their primary caregiver.

Parents of families in which the mother was diagnosed with cancer during or shortly after pregnancy mostly described no changes in their children. The reason, as the parents assumed, was that the disease of the mother represented normalcy to the children.

### Capabilities

Ill mothers and healthy fathers reported resources and coping strategies, which helped them to cope with the disease and the changed situation, on external, family and interpersonal level (Tables 3 and 5).

**Table 5:** Themes of resources including illustrative quotes

Themes	Quotes^a^
External resources (Mothers’ and fathers’ perspective)	
Support network (e.g. friends, peers, school)	• *“Your sister comes when she is needed, your brother, your father, and your mom is always there. I have to say, it was really nice to be able to say that we became close again. All of a sudden you get a lot of support.”* (Mother of two sons)
Competent medical staff	• *“And, of course, it is very helpful to have a doctor you can trust and who you can see eye to eye with.”* (Single mother of one son)
Flexible working hours	• *“Once he [husband] told his employer, he was given permission to leave anytime to support me. And he would just leave when he needed to.”* (Mother of two sons)
Financial security	• *“I’m always lucky to say that we are financially secure and that I, that we did not require any support during that time.”* (Mother of one daughter)
Children’s books or broschures	• *“I have this children’s book, […]. When I first read it I thought ‘what a horrible book, you can’t give this to a child.’ But then I said ‘okay, I will give it to her.’ She was so thrilled and wanted to have it read to her all the time. And it presented an opportunity to touch the subject. It was really great.”* (Mother of one daughter)
Family resources (Mothers’ and fathers’ perspective)	
Family time	• *“We did this trip together as a family. The weather was so warm and nice and we simply went to the beach, to this park by the beach. The children went into the water even though it was cold. It was so beautiful, and it still gives me comfort.”* (Mother of two children)
Children themselves	• *“He is such a cause for joy and brings a feeling of normalcy to my everyday life. And laughter, you just don’t sit stupidly on your couch and think about an uncertain future but instead build things with toy blocks. That adds to the fun, too. And he is just so precious.”* (Single mother of one son)
Open communication	• *“Because we are so open and everyone, including grandma, grandpa, teachers, and the neighbors know, you feel support from all directions.”* (Mother of two children)
Maintaining daily life	• *“Of course we try to not allow the illness to manage our life […] and, as far as possible, to continue to lead a normal one […]. We get up and have breakfast together […] the way it was when we both worked. One of us continues to drop her off and the other one picks her up. We try to maintain our regular routine. At night we generally let our daughter decide who tucks her in.”* (Father of one daughter)
Emotional support from partner and family/strong relationship	• *“My husband is really great. I am so lucky. He is always on my side and is supportive.”* (Mother of one son)
Family cohesion	• *“It is great to be able to rely on each other and to master a situation like this one in your marriage. Even though you know there were some bad times you also know that you could count on each other if push comes to shove. You can say that it created an even stronger bond, than just living day to day.”* (Mother of two children)
Flexible role division	• *“He has always been a great dad. When I had a hard time, well, we just complete each other. When I get home and see that he is in a tizzy I just grab the children and take them to the park or somewhere. And he does the same. When he noticed that it was starting again he would just leave me be and grab the children and do something with them.”* (Mother of two sons)
Intrapersonal resources (Mothers’ and fathers’ perspective)	
Time for oneself	• *“And now, after the children are dropped off at daycare, I can really appreciate being able to go downtown in the morning, at my own pace and just to look around. This was not really possible before.”* (Mother of two children)
Small aims for the future	• *“I think about the future, my career. I was already diagnosed with cancer when I took the exam, the final exam. Now I just have to write a dissertation at home. So I think that things will go on, which is rather motivating.”* (Mother of one son)
Self-efficacy	• *“She [the doctor] told me that while medicine is doing its part the rest is up to me and how I set my mind to it. That was very comforting since I am a thinker. I had the feeling that I was able to do something myself.”* (Single mother of two children)
Optimism/Positive attitude	• *“As a matter of fact, just because I believe that it will turn out well.”* (Father of two children)
Spirituality	• *“The right thoughts, faith plays a major role.”* (Mother of one daughter)

^a^original transcripts in German, quotes have been translated

#### External resources

All families used some kind of support network. Family members, e.g. grandparents or friends supported the families on days after chemotherapy and thus facilitated recuperation time for the mothers. Furthermore, the support network helped to keep up daily routines for the children. Moreover, families received institutionalized support such as day-care for the children or household help. Other institutionalized entities mentioned were social legal advice, teachers, physicians or psychologists.

Most healthy fathers reported that their employers released them from strict working hours and hence enabled fathers to accompany their partners to medical appointments or to take care for the children after day-care.

Many mothers described having a competent physician or nurse as a resource. Feeling that they got all relevant information about the disease and its treatment as well as getting answers to their medical questions helped the mothers to cope with the situation. Further, financial security due to sickness benefits or family savings was an important relieve for some families.

When it came to explaining the disease to the children, some parents reported using children’s books on parental illness or broschures on how to communicate with children about cancer.

#### Family resources and coping strategies

Most mothers and fathers reported that they recovered as a family when they spent time together. Some families described that in times when the mother felt well enough, parents and children made a day-trip or went for a short holiday. Other mothers reported that spending time together during daily routines was important and more intense.

Another important resource in face of the disease were the children themselves. Mothers as well as fathers described their children as a source of joy and happiness and the main cause to fight the disease.

Open communication with each other was important for many mothers and fathers to cope with the situation. Sharing feelings, concerns and fears with each other, but also open communication about the situation with their children, friends and extended family were considered helpful.

Maintaining daily life was described as coping strategy particularly for the children. Keeping up routines communicated that the disease could not disrupt family life and kept daily life as ‘normal’ as possible. Mothers and fathers described that they tried to minimize changes for their children to protect them from the impact of the disease.

Families supported each other emotionally and tried to encourage each other. Additionally they felt emotionally supported by their families and friends. Family cohesion was experienced as helpful, and some parents felt that the relationship with the children as well as with the partner grew even closer after diagnosis.

Fathers took over many tasks regarding household chores and child-care duties which had previously been performed by mothers. This flexible role division worked effortless in many families and reduced the mother’s responsibilities.

#### Intrapersonal resources and coping strategies

Particularly ill mothers reported that taking time for themselves was important for them to regain strength for the fight against the disease and for coping with the situation. They described trying to take time during the day to relax or to follow their hobbies.

Some mothers and fathers reported that they tried to look forward and set small aims e.g. in between chemotherapy cycles. They focused e.g. on family events in the future which let them look forward. Another resource some mothers and fathers described was self-efficacy. Believing that they were able to influence their situation helped them to feel in control and to master the situation.

Parents also reported that optimism and a positive attitude helped them to cope with the situation. Some ill mothers felt that their spirituality and faith helped them through the course of the disease.

## Discussion and conclusions

### Discussion

The results of this qualitative interview study substantiate many aspects that have been reported in the literature on the experiences of families affected by parental cancer. However, the inclusion of the healthy father’s perspective and the focus on demands and resources of the families provide a more comprehensive insight into the situation of affected families. Hence, detailed analyses of both strains and resources allow for more detailed conclusions on the supportive care needs of these families.

The ill mothers and healthy fathers reported changes through the cancer disease for themselves and for their children. They described a general impact of the disease, but also a specific impact on their parental role. In correspondence to findings of other studies [12, 22], parents described emotional but also practical issues such as taking care for the children after day-care or household duties as major challenges for the families. In our study, many families have children 5 years and younger, which may be a particularly challenging task for parents e.g. with regard to childcare. Mothers of babies and toddlers were emotionally distressed because they were concerned about the attachment to their children. Our study confirms the findings of other studies, that ill mothers are torn in their double role as patient and mother [6, 11] and experience fears and concerns about their children [4]. The results illustrate that all interviewed fathers were emotionally distressed and challenged by multiple burdens such as caring for the children, caring for the ill partner and occupational duties. This corroborates the findings of a previous study on the emotional distress of male partners of female cancer patients showing that they report higher levels of distress compared to fathers in the general population [23]. However, they need to be available because they often take on more responsibilities.

Single mothers are particularly burdened as they have no partner to lean on or may even additionally suffer from conflicts with the other parent. A current integrative review found single parenthood to be a significant predictor of poor adjustment and problems in children and adolescents [24].

According to the Family Adjustment and Adaptation Model the strains and stressors as well as the capabilities (resources, coping strategies) of the families influence the ability of adaptation to the situation. Our results show that after the cancer diagnosis all families used several resources or coping strategies. Supporting the results of other studies [6, 12], we identified practical support by a strong support network as an important resource. Support networks were necessary to enable parents to keep up daily routines for the children. These results are in accordance with other studies, which identified maintaining daily routines as an important coping strategy [11, 25]. In addition, intrapersonal resources such as optimism and self-efficacy seem to help parents to adjust to the situation. Additionally to competent medical staff, in particular fathers benefited from flexible working hours, which allowed them to take care for the children and their ill partner.

Parents experienced open communication with each other but also with their children as helpful to cope with the situation. Some parents used specific brochures or children’s books as a means to talk to their children. Families may benefit from professional support to facilitate communication between family members [26] but also to communicate with the extended family or institutions. This can help to activate the support network and reduce the parents’ burden. Besides flexible role division between parents, family cohesion and emotional support, parents described regaining strength when they spent time as a family. Offering day trips for affected families or activities such as ‘cancer-free time’ [27] seem to be a way to support families and to enhance family cohesion. However, one of the most important resources were the children themselves. Many mothers and fathers reported gaining strength and energy from their children.

Making use of resources may help families to cope with the exceptional circumstances caused by the mother’s cancer disease. The capabilities of the families may reduce the risk of maladaptation and may be a reason why many families successfully cope with the situation.

The availability of and the capability to make use of resources may be an indicator for the need of professional psychosocial support and counselling. In terms of the Family Adjustment and Adaptation Response Model, families may benefit from 1) a systematic assessment of their strains/stressors and resources/coping strategies and 2) professional support to reduce the strains/stressor where possible and enhance or activate family resources and coping strategies [9].

### Limitations

The results of this study cannot be generalized to all families of mothers with cancer due to the qualitative approach and the composition of the study sample. Many parents were highly educated and thus parents with lower education were underrepresented. One major limitation is that we were not able to include all healthy fathers due to organizational issues or refusal to contact the partner to prevent them from additional demands and duties. Since the consequences for healthy fathers when a mother has cancer are complex, we may not have reached thematic saturation for healthy fathers. Possibly, fathers who did not participate may be struggling even more and hence we may underestimate the burden of fathers.

Data analysis was based on the parental report with regard to the open interview questions and thus we cannot conclude how many parents actually experienced certain aspects. In two families, the ill mother and healthy father were interviewed together, because the families could not arrange two appointments. This constitutes another limitation of the study, since due to the mother’s presence the fathers may not have been as open about the stressors they experience and the responses of each parent may have been influenced by the response of the other parent. To avoid a selection bias regarding organizational issues, mothers and fathers could choose the interview setting. Hence, setting varied in terms of privacy (alone or with partner) and mode (in-person and via phone). This might bias the results with regard to openness of the interviewees or depth of the interviews [28].

We did not conduct any dyadic analyses with regard to similarities and differences between the father’s and the mother’s perspective. However, this would be an interesting element to consider in future studies.

### Conclusions

According to the FAAR Model [9], a major stressor such as a parental cancer diagnosis can lead to an imbalance of the demands of families and their capabilities to cope with the situation. Our findings indicate a high amount and diversity of stressors and strains for the ill as well as for the healthy parent and for the children. At the same time, families use diverse resources and coping strategies on external, family or intrapersonal level. To support mothers affected by cancer and their families, firstly, healthcare providers should be aware of the parental role of their patients. Furthermore, the results show that the healthy parents should also be involved as he or she might be the one maintaining daily life for the children to the extent possible while at the same time being in need for support themselves. Asking not only about burden and distress but also about existing and used resources may be the key to identify families in need for support. Activating resources and enhancing functional coping in the families may help them to adapt to the situation and protect them from maladjustment and adverse emotional consequences.

## Abbreviations

MaxQDA: Qualitative Data Analysis Software
UCCH: University Cancer Center Hamburg
